# The Influence of Age on Complications and Overall Survival After Ivor Lewis Totally Minimally Invasive Esophagectomy

**DOI:** 10.1007/s11605-018-4062-9

**Published:** 2018-12-18

**Authors:** Nikolaj S. Baranov, Frans van Workum, Jolijn van der Maas, Ewout Kouwenhoven, Marc van Det, Frits J. H. van den Wildenberg, Fatih Polat, Grard A. P. Nieuwenhuijzen, Misha D. P. Luyer, Camiel Rosman

**Affiliations:** 1 Department of Surgery, Radboud University Medical Center, Nijmegen, The Netherlands; 2 Department of Surgery, ZGT Hospital, Almelo, The Netherlands; 3 Department of Surgery, Canisius Wilhelmina Hospital, Nijmegen, The Netherlands; 4 Department of Surgery, Catharina Hospital, Eindhoven, The Netherlands

**Keywords:** Esophageal neoplasms, Esophagectomy, Aged, Postoperative complications, Mortality

## Abstract

**Background:**

The number of elderly patients suffering from esophageal cancer is increasing, due to an increasing incidence of esophageal cancer and increasing life expectancy. However, the effect of age on morbidity, mortality, and survival after Ivor Lewis total minimally invasive esophagectomy (TMIE) is not well known.

**Methods:**

A prospectively documented database from December 2010 to June 2017 was analyzed, including all patients who underwent Ivor Lewis TMIE for esophageal cancer in three Dutch high-volume esophageal cancer centers. Patients younger than 75 years (younger group) were compared to patients aged 75 years or older (elderly group). Baseline patient characteristics and perioperative data were included. Surgical complications were graded using the Clavien-Dindo scale. The primary outcome was postoperative complications Clavien-Dindo ≥ 3. Secondary outcome parameters were postoperative complications, in-hospital mortality, 30- and 90-day mortality and survival.

**Results:**

Four hundred and forty-six patients were included, 357 in the younger and 89 in the elderly group. No significant differences were recorded regarding baseline patient characteristics. There was no significant difference in complications graded Clavien-Dindo ≥ 3 and overall complications, short-term mortality, and survival. Delirium occurred in 27.0% in the elderly and 11.8% in the younger group (*p* < 0.001). After correction for baseline comorbidity this difference remained significant (*p* = 0.001). Median hospital length of stay was 13 days in the elderly and 11 days in the younger group (*p* = 0.010).

**Conclusions:**

Ivor Lewis TMIE can be safely performed in selected elderly patients without increasing postoperative morbidity and mortality.

## Introduction

The incidence of esophageal cancer is increasing, with a current incidence of 455,000 worldwide.^1^ The number of elderly patients diagnosed with esophageal cancer is increasing mainly due to an increased life expectancy of the general population. Esophageal resection with gastric conduit reconstruction after neoadjuvant therapy is the treatment of choice for patients with resectable esophageal carcinoma.^2, 3^ Esophagectomy is associated with considerable morbidity and mortality and this might be higher for elderly patients since they are more frail and generally have more comorbidities than younger patients.^4, 5^

Previous studies focusing on age-related morbidity and mortality regarding esophagectomy included patients that had been treated with open esophageal resection and showed that elderly patients have an increased peri-operative and in-hospital mortality risk, developed more postoperative complications and ultimately are confronted with a decreased 5-year survival rate.^6, 7^ Other studies, however, show no significant differences regarding short-term mortality and survival.^8, 9^ Nonetheless, most of these studies were single-center experiences, reported on small numbers of patients, included heterogeneous surgical approaches and very few underwent minimally invasive esophagectomy.^6–9^ Both a total minimally invasive esophagectomy (TMIE) and an Ivor Lewis approach have been shown to be associated with less postoperative morbidity compared with an open esophagectomy and a McKeown approach.^10–12^ Several studies regarding colon cancer have shown that laparoscopic assisted colectomy is associated with less postoperative morbidity when compared to open colectomy in elderly patients.^13, 14^ However, it is currently unknown whether or not a minimal invasive Ivor Lewis esophagectomy is also associated with acceptable morbidity for older patients.

Therefore, the objective of this study was to analyze the influence of age on short- and long-term outcomes after Ivor Lewis TMIE.

## Material and Methods

### Patients

All patients with esophageal carcinoma undergoing elective Ivor Lewis TMIE with curative intent were included. Patients aged ≥ 75 years (elderly group) were compared with patients < 75 years (younger group). The cutoff value of 75 years was chosen, because patients aged ≥ 75 years are generally excluded from clinical trials (including the TIME and MIRO trial).^10, 11^ Patients were operated on in three high-volume esophageal cancer centers in the Netherlands (Canisius-Wilhelmina hospital Nijmegen, Catharina hospital Eindhoven and ZGT hospital Almelo) from December 2010 to June 2017.

### Study Design

The three participating hospitals entered data of all esophageal cancer patients undergoing surgery into a prospective database. Data on patient-, tumor-, and operative characteristics, neoadjuvant treatment, postoperative complications, length of hospital and ICU stay, readmissions, postoperative mortality, pathology results, and overall survival were retrospectively analyzed.

### Outcome Parameters

The primary outcome parameter was complications graded ≥ III according to the Clavien-Dindo (CD) classification.^15^ Secondary outcome parameters were the associated failure to rescue (FTR), CD complications graded < II, in-hospital mortality, 30- and 90-day mortality, failure to rescue, and long-term survival.

### Definitions

Postoperative complications studied were pulmonary complications, cardiac complications, and other specified complications. Pulmonary complications were defined as the combined incidence of pneumothorax, pleural empyema, pleural effusion, a small group of other, less frequently diagnosed pulmonary complications called “other pulmonary complications,” and clinically diagnosed pneumonia defined according to the revised Uniform Pneumonia Score (r-UPS) for which treatment was started.^16^ Cardiac complications included atrial fibrillation, myocardial infarction, asystoly, and pericarditis, diagnosed by electrocardiogram, ultrasound, and/or laboratory findings. To account for cardiac complications that were not listed, and which are not as frequently diagnosed, those complications were combined in a small group of “other cardiac complications.” Other postoperative complications that were recorded included anastomotic leakage defined according to the Esophageal Complications Consensus Group (ECCG),^17^ delirium, jejunostomy (JJS)-related complications, urinary tract infection (UTI), urine retention, thrombo-embolic (TE) events, cerebrovascular accidents (CVA), chylothorax, and bronchoesophageal (BE) fistula.

Complications that could be directly attributed to the specific surgical process of an esophagectomy were considered to be “technical” and consisted of anastomotic leakage, chylothorax, and BE fistula. More general complications, occurring during hospital stay, were defined as “clinical” complications and included pulmonary complications, cardiac complications, delirium, CVA, UTI/urine retention, and TE events. In hospital, mortality was defined as any death occurring during hospital admittance. In addition, the 30-day, 90-day mortality, 1-year, and 2-year survival rates were analyzed.

### Operative Technique

Ivor Lewis TMIE consisted of laparothoracoscopic resection with intrathoracic esophagogastrostomy.^18^ Three types of intrathoracic anastomosis were used: 1) a linear stapled side-to-side (S-S) anastomosis, 2) a circular stapled end-to-side (E-S) anastomosis, or a handsewn end-to-end (E-E) anastomosis.

### Statistical Analysis

Statistical analysis was performed with the SPSS software package, version 24.0 (SPSS Inc., IBM Corporation Software Group, Somers, NY, USA). For dichotomous or ordinal variables, the Pearson’s chi-square test or Fisher’s exact test was used when appropriate. For non-normally distributed data, the Mann-Whitney *U* test was used. All tests were two-sided, and *p* values less than 0.05 were considered to be statistically significant. Binominal logistic regression analysis was used when differences in case mix parameters between the groups were associated with *p* values of < 0.1, to adjust for bias from differences in baseline patient characteristics. The Kaplan-Meier method was used to estimate the 1- and 2-year survival, with the Mantel-Cox log-rank test to determine whether there were statistically significant differences between the groups. In addition to the main analysis, hospital length of stay (LOS) was compared between elderly and younger patients in a subgroup that experienced complications and a subgroup that experienced severe complications, to evaluate whether there were any differences in how patients recover from complications.

## Results

Some 446 patients were enrolled and analyzed. Three hundred and fifty-seven patients were younger than 75 years (younger group) and 89 patients were aged 75 years and older (elderly group).

### Patient and Operation Characteristics

There was a trend towards a higher CCI score in the elderly group (*p* = 0.058, Table 1). ASA III tended to be higher in the elderly group (*p* = 0.063, Table 1). Other patient characteristics and operation characteristics did not differ significantly between the groups (Table 1 and Table 2).

**Table 1:** Patient characteristics

	Patients < 75 years	Patients ≥ 75 years	*p* value
	*N* = 357 (%)	*N* = 89 (%)	
Hospital			0.041
**–**1	148 (41.5%)	32 (36.0%)	
**–**2	163 (45.7%)	36 (40.4%)	
**–**3	46 (12.9%)	21 (23.6%)	
BMI			0.158
**–**Median/IQR	25.7 (5.4)	24.7 (3.8)	
Age			< 0.001
0.158**­**median/IQR	63.5 (10.3)	75.9 (3.3)	
Sex			0.625
**–**Male	293 (82.1%)	75 (84.3%)	
**–**Female	64 (17.9%)	14 (15.7%)	
ASA classification			0.063
**–**1	42 (11.8%)	5 (5.6%)	
**–**2	293 (66.9%)	56 (62.9%)	
**–**3	73 (20.4%)	28 (31.5%)	
**–**4	3 (0.8%)	0 (0.0%)	
Charlson Co-morbidity Index score (three groups)			0.058
**–**0	202 (56.6%)	38 (42.7%)	
**–**1	90 (25.2%)	28 (31.5%)	
**–**≥ 2	65 (18.2)	23 (25.8%)	
Neoadjuvant therapy			0.584
**–**Chemoradiotherapy	331 (92.7%)	80 (89.9%)	
**–**Chemotherapy	8 (2.2%)	2 (2.2%)	
**–**Radiotherapy	0 (0.0%)	0 (0.0%)	
**–**None	18 (5.0%)	7 (7.9%)	
Tumor stage			0.845*
**–**I	96 (26.9%)	25 (28.1%)	
**–**II	160 (44.8%)	40 (44.9%)	
**–**III	101 (28.3%)	22 (24.7%)	
**–**IV	0 (0.0%)	0 (0.0%)	
Tumor type			0.083
**–**SCC	49 (13.7%)	9 (10.1%)	
**–**Adenocarcinoma	300 (84.0%)	78 (87.6%)	
**–**Other	7 (2.0%)	0 (0.0%)	
**–**Unable to specify	1 (0.3%)	2 (2.2%)	
Tumor location			0.239
**–**Mid esophagus	25 (7.0%)	4 (4.5%)	
**–**Distal esophagus	253 (70.9%)	71 (79.8%)	
**–**Junction	79 (22.1%)	14 (15.7%)	
Anastomotic configuration			1.000
**–**End to end	4 (1.1%)	1 (1.1%)	
**–**End to side	177 (49.6%)	44 (49.4%)	
**–**Side to side	176 (49.3%)	44 (49.4%)	
Anastomotic technique			0.170
**–**Handsewn	31 (8.7%)	12 (13.5%)	
**–**Stapled	326 (91.3%)	77 (86.5%)	

*Patients from whom clinical stage could not be assessed were excluded. Total: 2

**Table 2:** Operation and pathology characteristics

	Patients < 75 years	Patients ≥ 75 years	*p* value
	*N* = 357 (%)	*N* = 89 (%)	
Abdominal conversion	7 (2.0%)	3 (3.4%)	0.425
Thoracic conversion	8 (2.2%)	3 (3.4%)	0.465
Operation time (min)			
–Median/IQR	252 (85)	278 (100)	0.064
Peri-operative blood loss (mL)			
–Median/IQR	100 (120)	100 (100)	0.938*
Lymph nodes			
–Median/IQR	21 (11)	20 (9)	0.424
R0-resection	340 (95.2%)	81 (91%)	0.121
Complete pathological response	60 (16.8%)	16 (18.0%)	0.793

*Patients from whom blood loss could not be assessed were excluded. Total: 18, 2 from the elderly group and 16 from the younger group

### Postoperative Outcome Parameters

Postoperative complications are shown in Table 3. There was no significant difference between the younger group and the elderly group regarding severe complications (35.9% in the younger group versus 43.8% in the elderly group, *p* = 0.421). Regarding the associated FTR, the younger group (8.6%) and the elderly group (11.1%) did not significantly differ (*p* = 0.743). The incidence of overall complications was 72.8% in the younger group versus 79.8% in the elderly group (*p* = 0.180). The incidence of technical complications was 24.6% in the younger group and 25.8% in the elderly group (*p* = 0.816) and clinical complications (69.7% versus 76.4%; *p* = 0.215). The incidence of cardiovascular complications was higher in the elderly group (14.0% versus 24.7%; *p* = 0.014). Other complications, reoperation rate, reintervention rate, and (ICU) length of stay did not significantly differ between the two groups. The incidence of postoperative delirium was 27% in the elderly group and 11.8% the younger group (*p* = <0.001). These differences persisted in binary logistic regression analyses correcting for comorbidity and ASA score. In addition, median hospital length of stay (LOS) was significantly shorter in the younger group than in the elderly group (11 versus 13 days, *p* = 0.010).

**Table 3:** Postoperative complications and length of stay

	Patients < 75 years	Patients ≥ 75 years	*p* value	Adjusted *p* value*	Odds (95%-BI)
	*N* = 357 (%)	*N* = 89 (%)			
Severe complications (CD ≥ 3)	128 (35.9)	39 (43.8)	0.421	0.625	1.13 (0.70, 1.83)
–FTR**	11 (8.6)	4 (11.1)	0.743	0.721	1.25 (0.37, 4.28)
Anastomotic leakage	61 (17.1)	17 (19.1)	0.654	0.715	1.12 (0.61, 2.03)
–Grade 1	5 (1.4)	3 (3.4)	0.201	0.256	2.32 (0.54, 9.95)
–Grade 2	33 (9.2)	8 (9.0)	0.941	0.881	0.94 (0.42, 2.12)
–Grade 3	23 (6.4)	6 (6.7)	0.918	0.899	1.06 (0.42, 2.70)
Chyle leakage	29 (8.1)	7 (7.9%)	0.936	0.940	0.97 (0.41, 2.29)
Esophagobronchial fistula	5 (1.4)	1 (1.1%)	1.000	0.658	0.61 (0.07, 5.44)
Pulmonary complications	222 (62.2)	56 (62.9)	0.989	0.979	0.99 (0.61, 1.61)
–Pneumonia	117 (32.8)	28 (31.5)	0.813	0.678	0.90 (0.54, 1.49)
–Empyema	42 (11.8)	10 (11.2)	0.889	0.863	0.94 (0.45, 1.96)
–Pneumothorax	58 (16.2)	11 (12.4)	0.364	0.320	0.70 (0.35, 1.41)
–Pleural effusion	93 (26.1)	26 (29.2)	0.546	0.550	1.17 (0.70, 1.96)
–Other	68 (19.0)	17 (19.1)	0.991	0.964	0.99 (0.55, 1.79)
Cardiac complications	50 (14.0%)	22 (24.7%)	0.014	0.019	1.98 (1.11, 3.50)
–AF	41 (11.5%)	17 (19.1%)	0.056	0.063	1.81 (0.97, 3.38)
–MI	2 (0.6%)	2 (2.2%)	0.179	0.124	4.79 (0.65, 35.20)
–Asystoly	5 (1.4%)	0 (0.0%)	0.588	0.997	0.00 (0.00, ∞)
–Pericarditis	1 (0.3%)	0 (0.0%)	1.000	0.997	0.00 (0.00, ∞)
–Other	5 (1.4%)	7 (7.9%)	0.003	0.003	6.09 (1.86, 19.90)
Other complications	99 (27.7)	39 (43.8)	0.003	0.006	1.98 (1.22, 3.20)
–Delirium	42 (11.8)	24 (27.0)	0.000	0.001	2.63 (1.48, 4.66)
–JJS related***	26 (7.3)	11 (12.4)	0.120	0.141	1.76 (0.83, 3.72)
–UTI/retention	10 (2.8)	4 (4.5%)	0.493	0.398	1.67 (0.51, 5.52)
–Trombo-embolic	8 (2.2)	2 (2.2%)	1.000	0.965	0.97 (0.20, 4.67)
–CVA	3 (0.8)	2 (2.2%)	0.261	0.250	2.91 (0.47, 18.04)
Total complication rate	260 (72.8)	71 (79.8)	0.180	0.228	1.42 (0.80, 2.51)
Technical complications	88 (24.6)	23 (25.8)	0.816	0.895	1.04 (0.61, 1.77)
Clinical complications	249 (69.7)	68 (76.4)	0.215	0.273	1.35 (0.79, 2.33)
Reintervention	114 (31.9)	33 (37.1)	0.355	0.425	1.22 (0.75, 1.98)
Reoperation	78 (21.9)	16 (18.0)	0.416	0.350	0.75 (0.41, 1.37)
Radiologic reintervention	48 (13.4)	8 (9.0)	0.256	0.253	0.63 (0.29, 1.39)
Endoscopic reintervention	47 (13.2)	18 (20.2)	0.095	0.087	1.70 (0.93, 3.12)
Re-intubation	34 (9.5%)	14 (15.7)	0.091	0.150	1.65 (0.83, 3.27)
ICU LOS (days)					
–Median/IQR	2 (3)	2 (5)	0.305		
ICU readmission	68 (19.0%)	17 (19.1%)	0.991	0.854	0.95 (0.52, 1.72)
Hospital LOS (days)					
–Median/IQR	11 (11)	13 (17)	0.010		
Hospital LOS > 21 days	81 (22.7)	28 (31.5)	0.085	0.112	1.52 (0.91, 2.54)
Hospital Readmission	58 (16.2%)	13 (14.6%)	0.705	0.561	0.82 (0.43, 1.59)

*p value adjusted for ASA and CCI score after binomial logistic regression analysis

**Younger group *N* = 128 and elderly group *N* = 39

***Jejunostomy-related complications

In both a subset of patients that experienced postoperative complications and a subset of patients that experienced severe complications, the median hospital LOS did not differ significantly between the elderly group and the younger group.

### Mortality and Survival

In-hospital mortality was 3.1% in the younger group versus 3.4% in the elderly group (*p* = 0.513) and 30-day mortality was 2.8% in the younger group versus 2.2% in the elderly group (*p* = 0.889). Ninety-day mortality was 5.0% in the younger group versus 9.0% in the elderly group (*p* = 0.155) (Table 4).

**Table 4:** Mortality and survival

	Patients < 75 years	Patients ≥ 75 years	*p* value	Adjusted *p* value*	Odds (95%-BI)
	*N* = 357 (%)	*N* = 89 (%)			
In-hospital mortality	11 (3.1)	4 (3.4)	0.513	0.591	1.38 (0.43, 4.48)
30-day mortality	10 (2.8)	2 (2.2)	1.000	0.699	0.74 (0.16, 3.46)
90-day mortality	18 (5.0)	8 (9.0)	0.155	0.208	1.75 (0.73, 4.20)
1-year survival	278 (77.9)	68 (76.4)	0.767	0.937	1.02 (0.59, 1.78)
2-year survival*	156 (54.5)	43 (57.3)	0.666	0.585	0.87 (0.52, 1.45)

*Patients < 75 years old *N* = 286; patients ≥ 75 years old *N* = 75

**Fig. 1:**
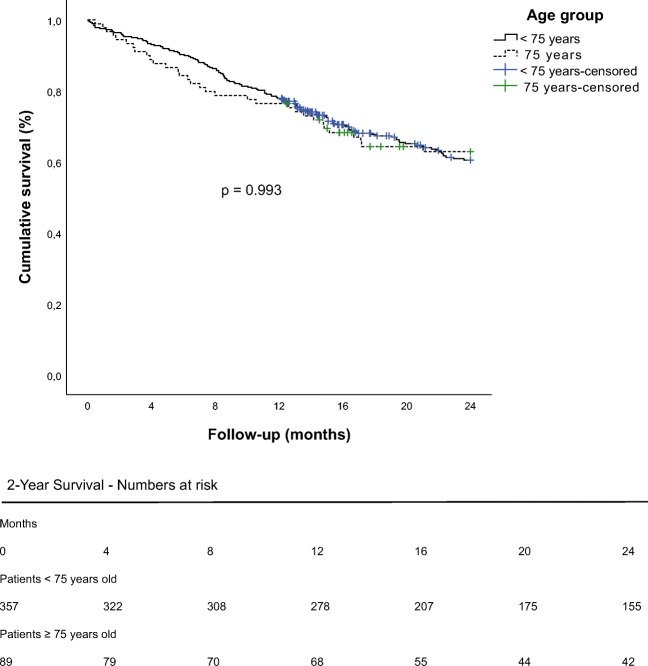
Two-year survival. Presented is an uncorrected Kaplan-Meier curve with a *p* value in the middle as the result from log-rank testing. The numbers-at-risk-table is presented below the curve

One-year survival was 77.9% in the younger group versus 76.4% in the elderly group (*p* = 0.767) and 2-year survival was 54.5% in the younger group and 57.3% in the elderly group (*p* = 0.666) (Fig. 1).

## Discussion

This study shows that the incidence of overall and severe complications (CD > 3) in this highly selected group of elderly patients undergoing Ivor Lewis TMIE is not higher than in younger patients. However, the incidence of cardiac complications and delirium were higher in the elderly group and hospital LOS was longer. Overall, this study supports the hypothesis that Ivor Lewis TMIE can be safely performed in selected elderly patients. Age alone should therefore not be a reason to withhold Ivor Lewis TMIE from patients.

The strengths of this study are, that this is one of the few studies investigating the influence of age on postoperative morbidity in a large multicenter outcome database and that this study specifically included patients undergoing Ivor-Lewis TMIE. In addition, we reported on a broad range of postoperative outcomes. Limitations of this study are its retrospective character and the fact that a relatively small number of elderly patients were included compared to younger patients, limiting statistical power. In addition, the cohort of elderly patients in our study is a very selected group, representing the healthiest patients in their population, which is also underlined by the limited difference in baseline comorbidity between elderly and younger patients. Unfortunately, none of the participating hospitals used objective and specified criteria for selecting the elderly patients for TMIE as well as the younger patients and, therefore, we were unable to determine what characteristics these patients were selected on, and what characteristics lead to other definitive, non-surgical treatment. Some elderly patients, initially deemed fit for surgery, may had to drop out before operation, because of toxicity side effects caused by the neoadjuvant chemoradiotherapy regimen. Unfortunately, we were unable to calculate the drop-out rates for both groups because data on drop-outs is not included in the surgical database. However, since drop-out rates after neoadjuvant chemoradiotherapy have been shown to be low in general (2–8%), it is unlikely that these drop-out rates significantly influenced our results.^2, 19^ More research is needed to investigate what criteria should be used for objectively selecting patients for curative esophageal surgery.

Studies on the influence of age in patients undergoing open esophagectomy show conflicting results. While some studies reported a higher incidence of non-surgical complications and higher mortality in the elderly group,^6, 7, 20^ other studies showed no difference in complications or mortality between the younger and elderly age groups.^8, 9^ For minimally invasive esophagectomy, a previous single-center study with 57 patients showed no significant difference in adverse events, length of stay, or mortality and which is grossly in line with the present study.^21^ Elderly patients may specifically benefit from minimally invasive esophagectomy, since it is associated with less postoperative morbidity compared to open esophagectomy.^10, 11^

The incidence of anastomotic leakage in the current study did not significantly differ between groups, but was higher than observed in previous studies.^21–24^ This may be attributed to the fact that, in the present study, all diagnosed anastomotic leaks were included, while others only reported anastomotic leaks requiring surgery representing only ECCG grade III leakages.^22^ The low percentage of ECCG grade III leakage as reported in the latter study (4.3%) was, however, comparable with our study (6.5%). Furthermore, the learning curve of minimally invasive surgical techniques may have played a role during the study period.^25, 26^

An important factor in the preoperative assessment of elderly patients is the presence of comorbidities, which have a large impact on the development of postoperative complications and overall survival after esophagectomy.^27^ This underlines the importance of assessing each potential candidate individually. In our study, the elderly group did not show a difference in comorbidities or ASA score compared with the younger group, which might be the result of the selection process. Although a longer hospital length of stay and a higher incidence of cardiac complications and delirium occurred in the elderly group, this observation did not result in a higher incidence of reinterventions, mortality, or impaired survival—which is in line with other studies.^28, 29^ Delirium, however, has a substantial negative impact on hospital length of stay, quality of life, and costs.^30^ It also increases the risk for institutionalization in a nursing home and dementia.^31^ However, the incidence still might be an underestimation, since delirium is often underdiagnosed.^32^ Several studies showed a positive effect on the incidence of postoperative delirium with the help of special hospital programs and preoperative screening.^33, 34^ One randomized trial showed that “geriatrics consultation reduced delirium by over one-third” adding to the argument that active participation of a geriatrician in the perioperative care process could be beneficial when dealing with elderly patients undergoing esophagectomy.

## Conclusion

Ivor Lewis TMIE can be safely performed in selected patients aged ≥ 75 years, without increasing severe complications or decreasing survival. Advanced age alone should not be a reason to withhold Ivor Lewis TMIE from patients. More research is needed to investigate what criteria should be used for selecting and preparing elderly patients with curable esophageal cancer for minimally invasive esophageal surgery.

## References

[1] Torre LA, Siegel RL, Ward EM, Jemal A (2016). Global Cancer Incidence and Mortality Rates and Trends–An Update. Cancer Epidemiol Biomarkers Prev.

[2] van Hagen P, Hulshof MCCM, van Lanschot JJB, Steyerberg EW, van Berge Henegouwen MI, Wijnhoven BPL (2012). Preoperative chemoradiotherapy for esophageal or junctional cancer. N Engl J Med.

[3] Cunningham D, Allum WH, Stenning SP, Thompson JN, Van de Velde CJH, Nicolson M (2006). Perioperative chemotherapy versus surgery alone for resectable gastroesophageal cancer. N Engl J Med.

[4] Koppert LB, Janssen-Heijnen MLG, Louwman MWJ, Lemmens VEPP, Wijnhoven BPL, Tilanus HW (2004). Comparison of comorbidity prevalence in oesophageal and gastric carcinoma patients: a population-based study. Eur J Gastroenterol Hepatol.

[5] Janssen-Heijnen MLG, Houterman S, Lemmens VEPP, Louwman MWJ, Maas HAAM, Coebergh JWW (2005). Prognostic impact of increasing age and co-morbidity in cancer patients: a population-based approach. Crit Rev Oncol Hematol.

[6] Cijs TM, Verhoef C, Steyerberg EW, Koppert LB, Tran TCK, Wijnhoven BPL (2010). Outcome of esophagectomy for cancer in elderly patients. Ann Thorac Surg.

[7] Tapias LF, Muniappan A, Wright CD, Gaissert HA, Wain JC, Morse CR (2013). Short and long-term outcomes after esophagectomy for cancer in elderly patients. Ann Thorac Surg.

[8] Alibakhshi A, Aminian A, Mirsharifi R, Jahangiri Y, Dashti H, Karimian F (2009). The effect of age on the outcome of esophageal cancer surgery. Ann Thorac Med.

[9] Ruol A, Portale G, Zaninotto G, Cagol M, Cavallin F, Castoro C (2007). Results of esophagectomy for esophageal cancer in elderly patients: age has little influence on outcome and survival. J Thorac Cardiovasc Surg.

[10] Biere SSAY, van Berge Henegouwen MI, Maas KW, Bonavina L, Rosman C, Garcia JR (2012). Minimally invasive versus open oesophagectomy for patients with oesophageal cancer: a multicentre, open-label, randomised controlled trial. Lancet Lond Engl.

[11] Mariette C, Meunier B, Pezet D, Dalban C, Collet D, Thomas P-A, et al. Hybrid minimally invasive versus open oesophagectomy for patients with oesophageal cancer: a multicenter, open-label, randomized phase III controlled trial, the MIRO trial. American Society of Clinical Oncology; 2015.

[12] van Workum F, Berkelmans GH, Klarenbeek BR, Nieuwenhuijzen GAP, Luyer MDP, Rosman C (2017). McKeown or Ivor Lewis totally minimally invasive esophagectomy for cancer of the esophagus and gastroesophageal junction: systematic review and meta-analysis. J Thorac Dis.

[13] Kazama K, Aoyama T, Hayashi T, Yamada T, Numata M, Amano S (2017). Evaluation of short-term outcomes of laparoscopic-assisted surgery for colorectal cancer in elderly patients aged over 75 years old: a multi-institutional study (YSURG1401). BMC Surg.

[14] Fujii S, Tsukamoto M, Fukushima Y, Shimada R, Okamoto K, Tsuchiya T (2016). Systematic review of laparoscopic vs open surgery for colorectal cancer in elderly patients. World J Gastrointest Oncol.

[15] Dindo D, Demartines N, Clavien P-A (2004). Classification of Surgical Complications. Ann Surg.

[16] Weijs TJ, Seesing MFJ, van Rossum PSN, Koëter M, van der Sluis PC, Luyer MDP (2016). Internal and External Validation of a multivariable Model to Define Hospital-Acquired Pneumonia After Esophagectomy. J Gastrointest Surg.

[17] Low DE, Alderson D, Cecconello I, Chang AC, Darling GE, DʼJourno XB (2015). International Consensus on Standardization of Data Collection for Complications Associated With Esophagectomy: Esophagectomy Complications Consensus Group (ECCG). Ann Surg.

[18] Lewis I (1946). The surgical treatment of carcinoma of the oesophagus; with special reference to a new operation for growths of the middle third. Br J Surg.

[19] Sathornviriyapong S, Matsuda A, Miyashita M, Matsumoto S, Sakurazawa N, Kawano Y (2016). Impact of Neoadjuvant Chemoradiation on Short-Term Outcomes for Esophageal Squamous Cell Carcinoma Patients: A Meta-analysis. Ann Surg Oncol.

[20] Poon RT, Law SY, Chu KM, Branicki FJ, Wong J (1998). Esophagectomy for carcinoma of the esophagus in the elderly: results of current surgical management. Ann Surg.

[21] Abbott A, Shridhar R, Hoffe S, Almhanna K, Doepker M, Saeed N (2015). Robotic assisted Ivor Lewis esophagectomy in the elderly patient. J Gastrointest Oncol.

[22] Luketich JD, Pennathur A, Awais O, Levy RM, Keeley S, Shende M (2012). Outcomes after minimally invasive esophagectomy: review of over 1000 patients. Ann Surg.

[23] Miyata H, Yamasaki M, Makino T, Miyazaki Y, Takahashi T, Kurokawa Y (2015). Clinical Outcome of Esophagectomy in Elderly Patients With and Without Neoadjuvant Therapy for Thoracic Esophageal Cancer. Ann Surg Oncol.

[24] Brown AM, Pucci MJ, Berger AC, Tatarian T, Evans NR, Rosato EL (2018). A standardized comparison of peri-operative complications after minimally invasive esophagectomy: Ivor Lewis versus McKeown. Surg Endosc.

[25] Tapias LF, Morse CR (2014). Minimally invasive Ivor Lewis esophagectomy: description of a learning curve. J Am Coll Surg.

[26] van Workum F, Stenstra MHBC, Berkelmans GHK, Slaman AE, van Berge Henegouwen MI, Gisbertz SS, et al. Learning Curve and Associated Morbidity of Minimally Invasive Esophagectomy: A Retrospective Multicenter Study. Ann Surg 2017. doi:10.1097/SLA.0000000000002469. 10.1097/SLA.0000000000002469 28857809

[27] Koppert LB, Lemmens VEPP, Coebergh JWW, Steyerberg EW, Wijnhoven BPL, Tilanus HW (2012). Impact of age and co-morbidity on surgical resection rate and survival in patients with oesophageal and gastric cancer. Br J Surg.

[28] Markar SR, Karthikesalingam A, Thrumurthy S, Ho A, Muallem G, Low DE (2013). Systematic review and pooled analysis assessing the association between elderly age and outcome following surgical resection of esophageal malignancy. Dis Esophagus Off J Int Soc Dis Esophagus.

[29] Takeuchi M, Takeuchi H, Fujisawa D, Miyajima K, Yoshimura K, Hashiguchi S (2012). Incidence and risk factors of postoperative delirium in patients with esophageal cancer. Ann Surg Oncol.

[30] Markar SR, Smith IA, Karthikesalingam A, Low DE (2013). The clinical and economic costs of delirium after surgical resection for esophageal malignancy. Ann Surg.

[31] Witlox J, Eurelings LSM, de Jonghe JFM, Kalisvaart KJ, Eikelenboom P, van Gool WA (2010). Delirium in elderly patients and the risk of postdischarge mortality, institutionalization, and dementia: a meta-analysis. JAMA.

[32] Fong TG, Tulebaev SR, Inouye SK (2009). Delirium in elderly adults: diagnosis, prevention and treatment. Nat Rev Neurol.

[33] Chen CC-H, Li H-C, Liang J-T, Lai I-R, Purnomo JDT, Yang Y-T (2017). Effect of a Modified Hospital Elder Life Program on Delirium and Length of Hospital Stay in Patients Undergoing Abdominal Surgery: A Cluster Randomized Clinical Trial. JAMA Surg.

[34] Watt J, Tricco AC, Talbot-Hamon C, Pham B, Rios P, Grudniewicz A (2018). Identifying older adults at risk of harm following elective surgery: a systematic review and meta-analysis. BMC Med.

